# FGF21 Reduces Lipid Accumulation in Bovine Hepatocytes by Enhancing Lipid Oxidation and Reducing Lipogenesis via AMPK Signaling

**DOI:** 10.3390/ani12070939

**Published:** 2022-04-06

**Authors:** Yezi Kong, Chenxu Zhao, Panpan Tan, Siqi Liu, Yan Huang, Fangyuan Zeng, Pingjun Ma, Yazhou Guo, Baoyu Zhao, Jianguo Wang

**Affiliations:** 1 College of Veterinary Medicine, Northwest A&F University, Xianyang 712100, China; kongyezi2019@nwafu.edu.cn (Y.K.); cxzhao@nwafu.edu.cn (C.Z.); tanpp@nwsuaf.edu.cn (P.T.); liusiqi@nwafu.edu.cn (S.L.); hy0016@nwafu.edu.cn (Y.H.); zengfy@nwafu.edu.cn (F.Z.); 2 Department of Physical Education, Northwest A&F University, Xianyang 712100, China; mpj8965@nwsuaf.edu.cn; 3 Department of Animal Engineering, Yangling Vocational and Technical College, Xianyang 712100, China; guoyazhou0819@163.com

**Keywords:** bovine hepatocytes, lipid metabolism, fibroblast growth factor 21, AMPK pathway, dairy cows

## Abstract

**Simple Summary:**

The negative energy balance (NEB) caused by decreased energy intake and increased demand during the perinatal period of dairy cows causes lipid mobilization, which leads to the accumulation of non-esterified fatty acids (NEFAs) in the liver. NEFAs are substrates that reduce triglyceride (TG) esterification synthesis, leading to energy metabolic disorders such as ketosis and fatty liver. Fibroblast growth factor 21 (FGF21) treatment can reduce TG content in the liver of dairy cows by 50%. This study further found that FGF21 regulated lipid metabolism in bovine liver cells through the AMPK signaling pathway. The expressions of SREBF1, the peroxisome proliferator-activated receptor α (PPARA) and their target genes were changed. This had a negative effect on lipid formation, enhanced lipid oxidation, and increased lipid transport. The present data suggest the possibility that FGF21 has potential value in alleviating perinatal metabolic diseases in dairy cows, and specific research in vivo should be studied in more detail.

**Abstract:**

During the periparturient period, dairy cows suffer drastic metabolic stress because of plasma increased non-esterified fatty acids (NEFAs) that stem from a negative energy balance. Fibroblast growth factor 21 (FGF21) is a hepatokine that activates the AMP-activated protein kinase (AMPK) signaling pathway to maintain intracellular energy balance and tissue integrity via the promotion of catabolism and the inhibition of anabolic regulation. FGF21 treatment caused a 50% reduction in triglyceride (TG) content in liver in dairy cows. However, it is not clear whether FGF21 regulates lipid metabolism in bovine liver. The purpose of this study was to evaluate the influence of FGF21 on lipid metabolism via AMPK signaling in bovine hepatocytes. The hepatocytes isolated from calves were treated with different concentrations of FGF21 or co-treated with AMPK inhibitor (BML-275). Herein, the study showed that FGF21 significantly reduced TG content in a dose–response manner and promoted very-low-density lipoprotein (VLDL) secretion via an up-regulation of the proteins (ApoB 100, ApoE and MTTP) involved in VLDL secretion. Otherwise, the genes associated with lipid transport (LDLR and CD36) and lipid oxidation (PPARGC1A, ACOX1 and CPT1A), were up-regulated following FGF21 treatment. Moreover, FGF21 treatment inhibited lipogenesis via SREBF1, ACACA, FASN and ACLY inhibition. After being co-treated with the AMPK inhibitor, FGF21-induced changes were reversed in some genes. In conclusion, these results indicate that FGF21 adaptively regulates energy metabolism for a negative impact on lipogenesis, strengthens lipid oxidation, and inhibited lipid transportation via AMPK signaling in bovine hepatocytes. The present data suggest the possibility that FGF21 has potential value in alleviating perinatal metabolic diseases in dairy cows, and specific research in vivo should be studied in more detail.

## 1. Introduction

The periparturient period (3 weeks before calving to 3 weeks after calving in cows) involves several physiological changes, as the body transitions from pregnancy to parturition, and eventually lactation [1]. During this period, because of a negative energy balance (NEB) due to a reduction in energy intake and an increase in requirement, large amounts of non-esterified fatty acids (NEFAs) are released into the plasma owing to lipid mobilization, resulting in the accumulation of NEFA in the liver [2]. As the liver does not have the oxidation capacity to handle excess NEFA, NEFA accumulates in this organ as triglyceride (TG). This causes liver-associated diseases, such as ketosis and fatty liver disease, which majorly hinder the development of the dairy and livestock industry [3]. In order to effectively manage such perinatal metabolic diseases in cows, NEFA oxidation needs to be increased and lipid accumulation in the liver controlled.

Fibroblast growth factor 21 (FGF21), a member of the fibroblast growth factor family, alleviates multifarious metabolic disorders such as metabolic syndrome, cardiovascular diseases, and atherosclerosis [4,5]. This hepatokine is primarily expressed in the liver and regulates glucose and lipid metabolism after fasting, ketogenic diet consumption, high-carbohydrate diet consumption, and exercise [6]. FGF21 increases glucose uptake into tissues by enhancing insulin sensitivity, thus decreasing serum hyperglycemia [7]. In mice, FGF21 knockdown stimulates glucose production by the liver [8]. In the fed state, transgenic mice with constitutive FGF21 expression in the liver have shown an increase in serum ketone bodies and a reduction in serum and hepatic TG levels [9]. FGF21 also increases the expression of enzymes involved in mitochondrial metabolism and stimulates the β-oxidation of fatty acids, thus improving mitochondrial respiratory capacity in adipocytes [10,11]. Therefore, FGF21 might be a probable regulator to improve hepatocyte ability to oxidize lipids for ameliorating metabolic disorders in perinatal dairy cows.

As an energy sensor that maintains the metabolic energy balance in tissues, AMP-activated protein kinase (AMPK) is involved in the signaling pathways via which FGF21 exerts its metabolic effects [12]. The activation of FGF21 and AMPK inhibits the transcriptional levels of the sterol regulatory element-binding transcription factor 1 (SREBF1) and the accumulation of TGs in hepatocytes [13,14]. AMPK activation suppresses the proteolytic processing and activation of SREBF1, further suppressing the expression of lipogenetic genes, reducing lipid deposition in the liver, and reducing the incidence of fatty liver disease [14,15]. SREBF1 is a negative regulator of FGF21, and FGF21 inhibits fat synthesis by down-regulating the transcription of SREBF1 and destroying the molecular machinery required for SREBF1 processing and maturation [13]. Therefore, FGF21 might regulate liver lipid metabolism through AMPK in dairy cows.

In healthy perinatal cows, FGF21 and NEFA concentrations in the blood were correlated with each other [16]. Moreover, in cows with fatty liver disease and ketosis, the FGF21 concentration in blood was significantly higher than that in a matched population of healthy cows [16]. In vitro experiments showed that the NEFAs significantly promoted the expression and activity of FGF21 in bovine hepatocytes, suggesting that the expression of FGF21 was stimulated owing to the NEB [17]. The important target tissues of FGF21 are liver and adipose tissue. FGF21 reduces the ability of lipid accumulation in bovine liver by reducing the inflow of adipose tissue-derived fatty acids at super-physiological doses [18]. In rodents, FGF21 increases fatty acid oxidation in the liver and inhibits lipolysis in adipose tissue. This activation process can be mediated by AMPK [18]. Therefore, we hypothesized that FGF21 might have a positive effect on improving liver lipid accumulation via AMPK signaling in dairy cows. The present study aimed to investigate the effect of FGF21 on lipid metabolism through AMPK signaling in bovine hepatocytes.

## 2. Materials and Methods

### 2.1. Isolation, Culture of Hepatocytes, and FGF21 Treatment

After anesthetic treatment using thiamylal sodium, the caudate liver lobe of newborn calves was obtained under aseptic conditions within 24 h. Subsequently, hepatocytes were isolated using a modified 2-step collagenase perfusion method, as described previously [19,20]. Perfusion A (140 mmol/L NaCl, 6.7 mmol/L KCl, 10 mmol/L HEPES, 0.5 mmol/L EDTANa_2_ and 2.5 mmol/L glucose; pH 7.2–7.4, 37 °C), perfusion B (140 mmol/L NaCl, 6.7 mmol/L KCl, 30 mmol/L HEPES, 5 mmol/L CaCl_2_, and 2.5 mmol/L glucose; pH 7.2–7.4, 37 °C), and perfusion C (0.2 g/L collagenase IV (Gibco; Gaithersburg, MD, USA) in perfusion B, pH 7.2–7.4, 37 °C) were used [21,22]. After terminating collagenase digestion, the tissue was cut into smaller pieces to release the hepatocytes. Blood vessels and connective tissue were removed, and a tissue suspension was obtained. This suspension was filtered through 100-mesh (150 µm) and 200-mesh (75 µm) cell sieves. The obtained hepatocyte suspension was then centrifuged for 5 min at 500× *g* at 4 °C (Sigma; St Louis, MS, USA) and washed twice in RPMI-1640 basic medium (31800022, Gibco; Gaithersburg, MD, USA). Cell viability was assessed using the Trypan blue dye exclusion method. The hepatocytes were used to perform further experiments if the percentage of viable cells was close to at least 95%. The cells were adjusted to 1.5 × 10^6^ cells/mL with adherent culture medium consisting of 10% calf serum, 10^−6^ mol/L insulin, 10^−6^ mol/L DEX, 10 μg/mL Vc, 100 U/mL benzylpenicillin, and 100 μg/mL streptomycin. The hepatocytes were seeded into 6-well culture plates that were stored at 37 °C in 5% CO_2_. After 4 h, the medium was replaced with growth medium containing 10% fetal bovine serum (FB15015, Clark Bioscience; Richmond, VA, USA), which was changed with fresh growth medium every 24 h for three days.

After that, cells were treated with recombinant bovine FGF21 (greater than 98.0%; CYT-657, Prospec; Rehovot, Israel). According to our previous study, the dosage in blood range during the peripartum period in cow ranges from 300 to 1800 pg/mL, and the content of FGF21 in cell supernatant was close to 900 pg/mL after 2.4 mmol/L NEFA treatment [16]. Therefore, the hepatocytes were treated with 900 pg/mL FGF21 for 0, 2, 4, 8, 12 and 24 h. For the dose–response experiments, the hepatocytes were divided into the following six groups: 0 pg/mL FGF21 (control), 300 pg/mL FGF21 (lowest), 900 pg/mL FGF21 (medium), 1800 pg/mL FGF21 (highest), 10 μmol/L BML-275 (AMPK inhibitor; HY-13418A, MCE; Malta, NY, USA), and 900 pg/mL FGF21 + 10 μmol/L BML-275.

### 2.2. Oil Red O Staining of Lipids in Hepatocytes

Oil red O staining was performed according to the manufacturer’s instructions (Solarbio ORO Red Staining, Beijing Solarbio Science & Technology Co., Ltd.; Beijing, China). Briefly, after washing twice with 0.01 mol/L PBS, the hepatocytes were fixed with 4% paraformaldehyde for 30 min and then washed twice with ddH_2_O. The cells were incubated with 60% isopropanol for 5 min. Freshly prepared ORO stain was added and the cells were placed in the staining solution for 15 min, before being washed five times with ddH_2_O. Cell nuclei were counterstained with a Mayer hematoxylin staining solution for 2 min and then washed five times with ddH_2_O. ORO buffer was added for 1 min and distilled water was added to cover the cells. The cells were then observed under a light microscope (Leica Microsystems; Wetzlar, Germany). Four images were selected to calculate the percentage of stained area of interest using Image Pro plus (Media Cybernetics; Silver Spring, MD, USA), and finally the control group was used for normalization.

### 2.3. Determination of Extracellular VLDL and Intracellular TG Content

After treatment, the supernatant from the cell culture was collected to determine the content of VLDL with the bovine VLDL ELISA Kit (E11V0006, BlueGene Biotech; Shanghai, China) using the manufacturer’s instructions. 

The hepatocytes were harvested into centrifuge tubes and washed twice with ice-cold 0.01 mol/L PBS, before the lysis step with 0.1 mL 1% Triton X-100. The TG content was determined using the manufacturer’s protocol of TG Assay Kit (Nanjing Jianchen; Nanjing, China).

### 2.4. RNA Extraction and qRT-PCR Analysis

Total hepatic RNA was isolated from hepatocytes using the TRIzol reagent (Invitrogen; Carlsbad, CA, USA) according to the manufacturer’s instructions. When the OD260/280 was between 1.8 and 2.0, the isolated RNA was reverse-transcribed into cDNA using the manufacturer’s instructions (Fast Quant RT Kit, Tian Gen; Beijing, China). Then, mRNA expression was determined with qRT-PCR using the SYBR Green QuantiTect qRT-PCR Kit (TaKaRa Biotechnology Co., Ltd.; Tokyo, Japan). All qRT-PCR tests were performed in triplicate on an ABI Prism 7900HT system (Applied Biosystems), and results were normalized based on 18S levels. The gene primers were designed using Primer 6.0 (PE Applied Biosystems Inc., Foster, CA, USA) and are shown in Table 1. The amplification efficiency of the primers ranged from 0.9 to 1.05.

### 2.5. Immunofluorescence Staining

The hepatocytes were fixed with 4% paraformaldehyde for 30 min and washed thrice with 0.01 mol/L PBS. The hepatocytes were then incubated in a solution containing 5% goat serum and 0.1% Triton X-100 (Sigma; St Louis, MS, USA), along with antibodies against SREBF1 (1:100, OM121949, OmnimAbs; Alhambra, CA, USA), and PPARA (1:100, PR-4003, HopeBiot; Zhenjiang, China) overnight at 4 °C. After that, the cells were washed five times with 0.01 mol/L PBS. Following this, the hepatocytes were incubated with secondary antibodies conjugated to fluorescent labels (ab150073, Abcam; Cambridge, UK) and 4′,6-diamidino-2-phenylindole (DAPI, D9542, Sigma; St Louis, MS, USA) for 4 h at room temperature. After being washed five times with 0.01 mol/L PBS, the cells were observed under a laser confocal microscope (Carl Zeiss; Jena, Germany).

### 2.6. Western Blot Analysis

After treatment, total cellular and nuclear proteins were extracted according to the M-PER^®^Mammalian Protein Extraction Reagent and NE-PER^®^Nuclear and Cytoplasmic Extraction Reagents instruction manual (Thermo; Waltham, MA, USA), respectively. After denaturation, the proteins were resolved using SDS-PAGE and transferred to a PVDF membrane. The membranes were blocked with blocking solution and incubated overnight at 4 °C with primary antibodies against AMPK (1:1000, 5832T, CST; Danvers, MA, USA), p-AMPK (1:1000, AA393, Beyotime; Shanghai, China), LKB1 (1:1000, PB0257, Boster; Wuhan, China), SREBF1 (1:1000), PPARA (1:1000), peroxisome proliferator-activated receptor γ coactivator 1α (PPARGC1A, 1:1000, ab54481, Abcam; Cambridge, UK), sirtuin 1 (SIRT1, 1:1000, AF0282, Beyotime; Shanghai, China), fatty acid synthase (FASN, 1:1000, D262701, BBI Life Science; Shanghai, China), stearoyl-coenzyme A desaturase-1 (SCD1, 1:1000, ab23331, Abcam; Cambridge, UK), ATP citrate lyase (ACLY, 1:1000, BM4399, Boster; Wuhan, China), acetyl coenzyme A carboxylase 1 (ACACA, 1:1000, 3662, CST; Danvers, MA, USA), p-ACACA (1:1000, AA110, Beyotime; Shanghai, China), peroxisomal acyl-coenzyme A oxidase 1 (ACOX1, 1:1000, 10957-1-AP, Proteintech; Chicago, IL, USA), carnitine palmitoyltransferase 1α (CPT1A, 1:1000, ab83862, Abcam; Cambridge, UK), carnitine palmitoyltransferase 2 (CPT2, 1:1000, AF2356, Beyotime; China), microsomal triglyceride transfer protein (MTTP, 1:1000, D154124, BBI Life Science; Shanghai, China), low-density lipoprotein receptor (LDLR, 1:1000, AF1438, Beyotime; Shanghai, China), liver-fatty acid-binding protein (L-FABP, 1:1000, PB0644, Boster; Wuhan, China), fatty acid transporter CD36 (CD36, 1:1000, PB0398, Boster; Wuhan, China), apolipoprotein B100 (ApoB 100, 1:1000, PB1096, Boster; Wuhan, China), and apolipoprotein E (ApoE, 1:1000, AF1921, Beyotime; Shanghai, China). After adequate washing, membranes were incubated with horseradish peroxidase-conjugated anti-rabbit or anti-mouse IgG (1:5000, A0208 and A0216, Beyotime; Shanghai, China) at room temperature for 2 h. Finally, the bands were visualized using an enhanced chemiluminescence reagent (Beyo ECL Plus, Beyotime; Shanghai, China). Protein gray intensity was quantified by Image-Pro Plus 6.0 (Media Cybernetics, Bethesda, MD, USA), and normalized to β-actin or LamA/C.

### 2.7. Statistical Analysis

Each experiment was repeated at least three times. The results were expressed as the mean ± standard deviation (SD) and illustrated using GraphPad Prism 7 (GraphPad Software Inc., San Diego, CA, USA). The differences among groups were compared using a one-way ANOVA and Duncan’s multiple range test. *p* < 0.05 indicates statistical significance.

## 3. Results

### 3.1. FGF21 Stimulates Hepatocellular VLDL Secretion, Resulting in a Reduction in Intracellular TG Content

**Figure 1:**
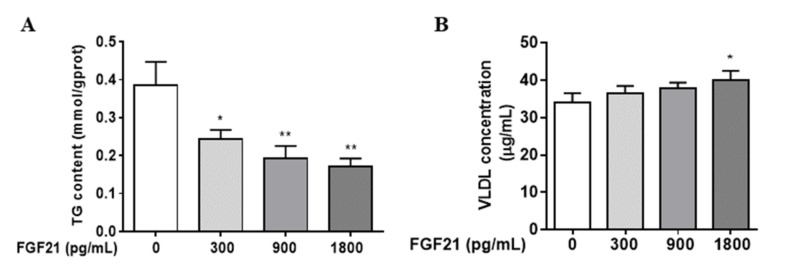
FGF21 stimulates hepatocellular VLDL secretion, resulting in the reduction in intracellular TG content. The content of intracellular TG (**A**) and extracellular VLDL (**B**) were detected after being treated with different concentrations of FGF21 for 24 h in primary hepatocyte. Three independent experiments were performed. Statistically significance was indicated: *, *p* < 0.05; **, *p* < 0.01, * was compared with control group.

Firstly, we examined whether FGF21 affected hepatic TG content and VLDL secretion. As shown in Figure 1, after treatment with different concentrations (0, 300, 900, 1800 pg/mL) of FGF21 for 24 h, hepatocellular TG concentrations decreased in a dose-dependent manner. The decrease observed after 900 and 1800 pg/mL treatment was about 50% and 65%, respectively (*p* < 0.01). FGF21 enhanced 18% of the extracellular concentration of VLDL at 1800 pg/mL (*p* < 0.05).

### 3.2. FGF21 Enhances AMPK Phosphorylation

**Figure 2:**
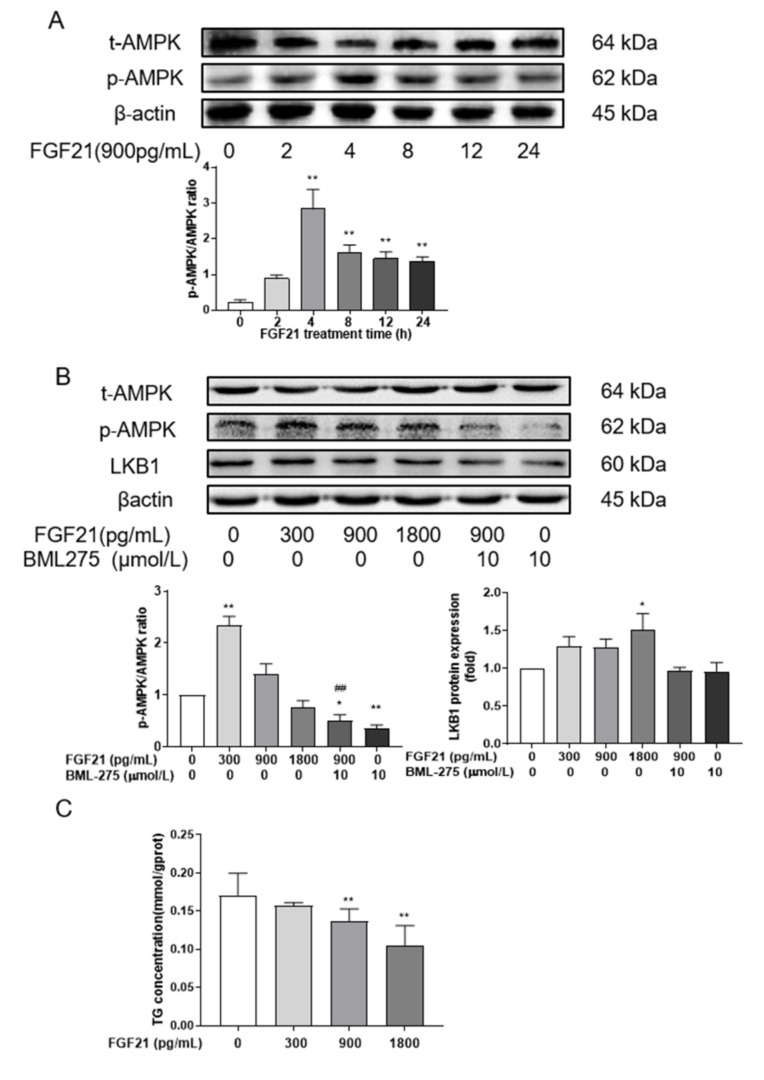
FGF21 regulates lipid accumulation by modulating AMPK phosphorylation. Time (**A**) and dose (**B**) effects of FGF21 on AMPK phosphorylation in hepatocytes; the content of intracellular TG (**C**) was detected after being treated with FGF21 for 4 h. Three independent experiments were performed. Statistically significance was indicated: *, *p* < 0.05; ** and ^##^, *p* < 0.01; * was compared with control group, ^#^ was compared with 900 pg/mL group.

To explore whether FGF21 regulated lipid accumulation by modulating AMPK activity, the levels of p-AMPK in hepatocytes were detected. The results showed that the phosphorylation of AMPK was increased at all time points after FGF21 treatment, with an extremely significant difference at 4, 8, 12 and 24 h (Figure 2A). The phosphorylation of AMPK fluctuated with time, but it peaked at 4 h.

Subsequently, the effect of different concentrations of FGF21 was examined at 4 h. It was found that the p-AMPK/AMPK ratio was the highest with 300 pg/mL FGF21 treatment (*p* < 0.01), but there were no differences in this ratio after treatment with 900 and 1800 pg/mL FGF21 when compared with the control group (*p* > 0.05, Figure 2B). After adding the AMPK inhibitor BML-275, the level of AMPK phosphorylation became remarkably lower than that observed with control treatment. However, the phosphorylation of AMPK was not different between FGF21 combined with the BML-275 group and the BML-275 group, and was significantly lower than the 900 pg/mL FGF21 group (*p* < 0.01). Similarly, the expression of LKB1, a kinase upstream of AMPK, was induced after FGF21 treatment.

The changes in primary hepatocellular TG were also detected at 4 h post-treatment. As shown in Figure 2C, FGF21 had a significant inhibitory effect on the concentration of TG in a dose-dependent manner. With 1800 pg/mL FGF21 treatment, TG levels decreased 40% (*p* < 0.01).

### 3.3. FGF21 Inhibits Lipid Synthesis by Reducing SREBF1 Nuclear Entry and Increasing ACACA Phosphorylation Ratio

**Figure 3:**
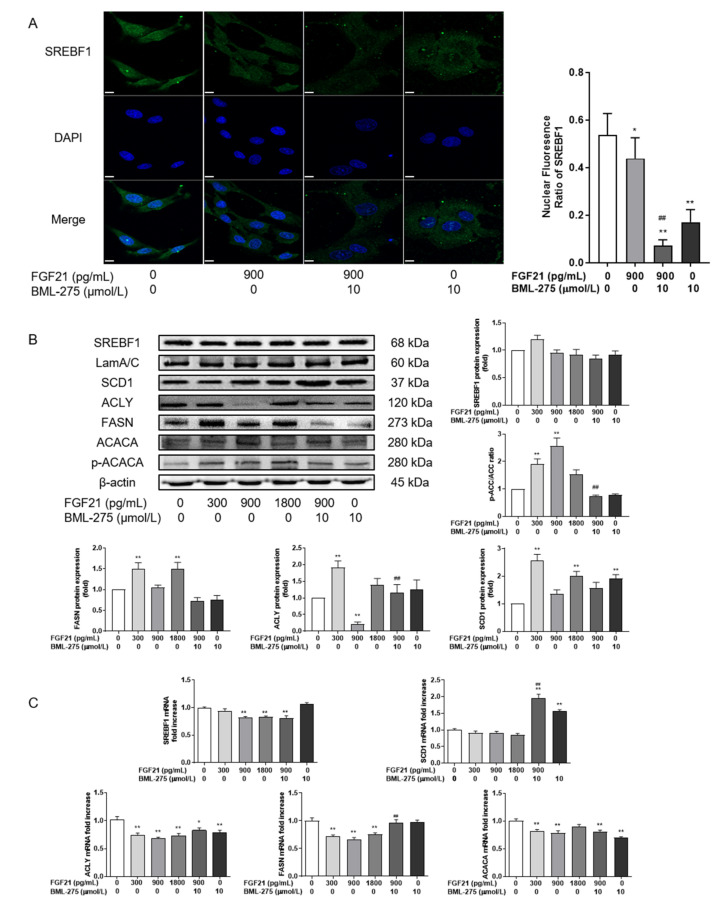
FGF21 inhibits lipid synthesis by reducing SREBF1 nuclear entry and increasing the ACACA phosphorylation ratio. Hepatocytes were treated with FGF21 and BML-275 individually or in combination for 4 h. (**A**) Immunofluorescence staining was conducted to determine the effect of FGF21 or BML-275 on SREBF1 nuclear translocation in hepatocytes (**left**). Green, SREBF1 staining; blue, DAPI nuclear staining (630×). Fluorescent intensity from 4 randomly selected microscopic fields per group (**right**). (**B**) Western blot analysis of SREBF1, FASN, SCD1, ACLY, p-ACACA and ACACA. (**C**) Relative mRNA expression levels of SREBF1, FASN, SCD1, ACLY and ACACA. Three independent experiments were performed. Statistical significance was indicated: *, *p* < 0.05; ** and ^##^, *p* < 0.01; * was compared with control group, ^#^ was compared with 900 pg/mL group.

In order to investigate whether the decrease in the TG content was caused by reduced lipid synthesis, the expression of the SREBF1 protein was evaluated against its downstream targets FASN, SCD1, ACLY and ACACA (Figure 3). These results showed that FGF21 reduced SREBF1 nuclear entry (*p* < 0.05) compared to no FGF21 treatment (Figure 3A). SREBF1 nuclear entry was also reduced after AMPK activity inhibition. After the combined use of the FGF21 and AMPK inhibitor, the inhibitory effect was enhanced (*p* < 0.01). The expression results of SREBF1 and its target genes showed that FGF21 had little effect on SREBF1 protein expression compared with the control group. At the concentrations of 300 and 1800 pg/mL, it could increase the FASN and SCD1 protein expression of de novo lipogenesis (*p* < 0.01, Figure 3B). However, 900 pg/mL of FGF21 did not affect the levels of these proteins, and neither did the combination with BML-275. It should be noted that the ACACA phosphorylation ratio was significantly increased after FGF21 treatment, especially at 900 pg/mL, which increased by about 2.5 times (*p* < 0.01, Figure 3B). ACLY was also decreased by 50% (*p* < 0.01). FGF21 could significantly inhibit the mRNA levels of SREBF1 and its target genes compared with the control group, and the difference was extremely significant at 900 pg/mL FGF21 (*p* < 0.01). When combined with FGF21 and BML-275, the abundance of SREBF1, ACLY and ACACA mRNA was significantly reduced, compared with the control group; on the contrary, the difference was not significant when compared with 900 pg/mL FGF21.

### 3.4. FGF21 Promotes the Expression of Key Proteins in Intracellular Fatty Acid Oxidation

**Figure 4:**
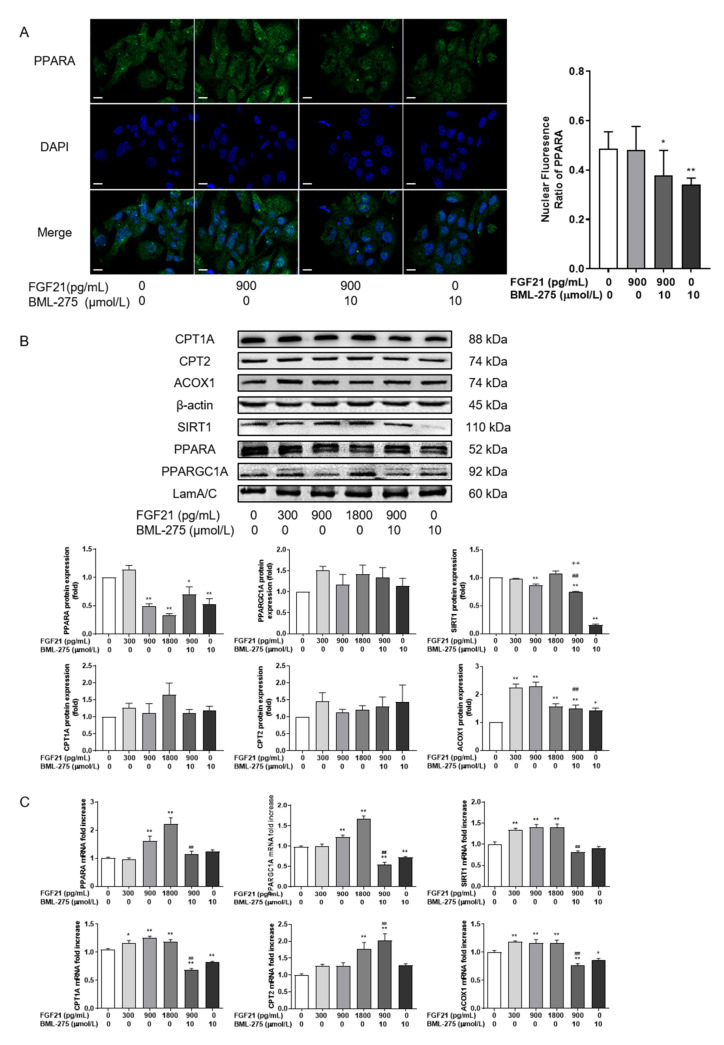
FGF21 promotes the expression of key proteins in intracellular fatty acid oxidation. Hepatocytes were treated with FGF21 and BML-275 individually or in combination for 4 h. (**A**) Immunofluorescence staining was conducted to determine the effect of FGF21 or BML-275 on PPARA nuclear translocation in hepatocytes (**left**). Green, PPARA staining; blue, DAPI nuclear staining (630×). Fluorescent intensity from 4 randomly selected microscopic fields per group (**right**). (**B**) Western blot analysis of PPARA, CPT1A, CPT2, ACOX1, PPARGC1A and SIRT1. (**C**) Relative mRNA expression levels of PPARA, CPT1A, CPT2, ACOX1, PPARGC1A and SIRT1. Three independent experiments were performed. Statistical significance was indicated: *, *p* < 0.05; **, ^++^ and ^##^, *p* < 0.01; * was compared with control group, ^#^ was compared with 900 pg/mL group, ^+^ was compared with BML-275 group.

Then, the expressions of PPARA were evaluated, the main regulators of lipid oxidation in hepatocytes, as well as their target genes ACOX1, CPT1A, and CPT2 (Figure 4). Immunofluorescence labelling showed that PPARA was translocated further into the nucleus after 900 pg/mL FGF21 treatment than the control group with no significant difference, and BML-275 remarkably prevented the translocation of PPARA to the nucleus. There was little difference between FGF21 in combination with BML-275 and FGF21 alone (Figure 4A). 

The results of protein levels of PPARA and its target genes showed that although the content of PPARA was significantly decreased after 900 and 1800 pg/mL FGF21 treatment, CPT1A, CPT2 and ACOX1 were increased to varying degrees, with a significant increase only in ACOX1 (*p* < 0.01, Figure 4B), compared with the control group. At the concentration of 300 pg/mL, protein levels were increased to varying degrees for PPARA, CPT1A, CPT2 and ACOX1. Moreover, the ACOX1 was increased two-fold compared to the control group. The content of the PPARA, CPT1A and CPT2 proteins had no significant effect on the changes compared with 900 pg/mL, but could significantly reduce the expression of ACOX1 at 900 pg/mL + BML-275 group. At the transcriptional level (Figure 4C), the expression of PPARA and its target genes increased at 1800 pg/mL compared with the control group. Moreover, there were significant changes in CPTA and ACOX1 at 300 pg/mL. The mRNA content of PPARA, CPT1A, and ACOX1 was reduced for combined with BML-275 when compared with using 900 pg/mL FGF21 alone (*p* < 0.01).

At the same time, we also detected the potential mitochondrial biogenesis. The results showed that this could slightly affect the protein level of PPARGC1A and its upstream protein SIRT1 (*p* > 0.05) at 1800 pg/mL FGF21 treatment when compared with the control group (Figure 4B), while their transcript levels were both increased after FGF21 treatment compared with the control group (Figure 4C). A significant increase in PPARGC1A was reached at 900 pg/mL, and SIRT1 at 300 pg/mL. The mRNA levels of PPARA and SIRT1 were significantly lower after the combination with BML-275 compared to treatment with 900 FGF21, as well as the protein levels of SIRT1.

### 3.5. FGF21 Promotes Lipid Transport and Increases the Secretion of VLDL

**Figure 5:**
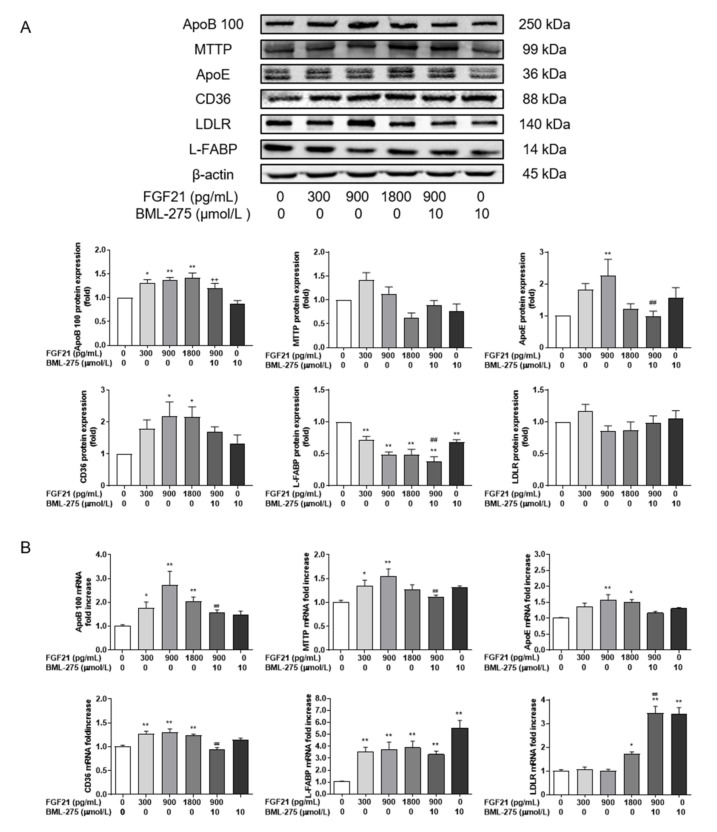
FGF21 promotes lipid transport and increases the secretion of VLDL. Hepatocytes were treated with FGF21 and BML-275 individually or in combination for 4 h. Western blot analysis of ApoB 100, ApoE, MTTP, CD36, LDLR and L-FABP (**A**) and relative mRNA expression levels of ApoB 100, ApoE, MTTP, CD36, LDLR and L-FABP. (**B**). Three independent experiments were performed. Statistical significance was indicated: *, *p* < 0.05; **, ^++^ and ^##^, *p* < 0.01; * was compared with control group, ^#^ was compared with 900 pg/mL group, ^+^ was compared with BML-275 group.

Next, the expression of MTTP, ApoB 100 and ApoE, the main proteins involved in VLDL assembly and secretion, was evaluated (Figure 5A). FGF21 significantly elevated the expression of ApoB 100 compared with control group (*p* < 0.05). ApoE showed a significant increase at 1800 pg/mL (*p* < 0.01). Only ApoE decreased markedly in the group of FGF21 + BML-275, compared to 900 pg/mL FGF21. FGF21 increased the mRNA abundance of these genes (Figure 5B), peaking at 900 pg/mL (*p* < 0.01). The mRNA levels of ApoB 100 and MTTP were significantly reduced at FGF21 combined with the BML-275group, compared with the 900 pg/mL group.

To probe the effect of AMPK signaling on lipid transport, the expression of lipid transport-related proteins was evaluated. It was found that FGF21 significantly increased the protein expression of CD36 (*p* < 0.01) and had no difference in the effect on LDLR (*p* > 0.05) when compared with the control group, while L-FABP was significantly inhibited (*p* < 0.01, Figure 5A). L-FABP protein expression decreased at 900 pg/mL FGF21 (*p* < 0.01) when compared with the combined treatment group, while CD36 and LDLR expression did not become higher (*p* > 0.05). Regarding to mRNA levels (Figure 5B), the abundance of L-FABP mRNA was increased more than three-fold with different FGF21 concentrations (*p* < 0.01), with a significant increase in CD36 mRNA (*p* < 0.01), compared with the control group. LDLR mRNA was significantly increased at 1800 pg/mL only, whereas the mRNA levels of CD36 increased for FGF21 treatment (*p* < 0.01) compared with the co-treatments of FGF21 and BML-275, but the LDLR mRNA change was lesser in the FGF21 group (*p* < 0.01).

**Figure 6:**
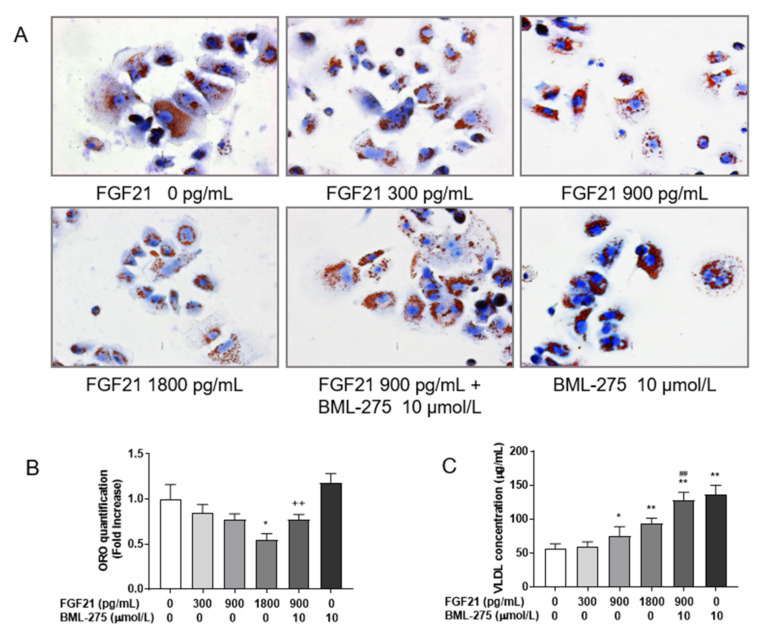
FGF21 decreases ORO staining area and increases the secretion of VLDL in hepatocytes. (**A**) Oil red staining of hepatocytes treated with FGF21 and BML-275 individually or in combination for 4 h (400×). (**B**) Quantitative analysis after ORO staining from 4 randomly selected images, normalized by the control group. (**C**) The content of cell supernatant VLDL was detected after treated with FGF21 and BML-275 individually or in combination for 4 h. Statistical significance was indicated: *, *p* < 0.05; **, ^++^ and ^##^, *p* < 0.01; * was compared with control group, ^#^ was compared with 900 pg/mL group, ^+^ was compared with BML-275 group.

In order to observe the effect of different concentrations of FGF21 on lipid droplets, treated cells were observed using ORO staining to evaluate cell morphology (Figure 6A). With increasing concentrations of FGF21, the number and size of lipid droplets in the cells stained orange–red reduced, and these droplets became more concentrated around the nucleus. Quantitative analysis showed that the lipid droplet staining area decreased in a dose-dependent manner compared with the control group (Figure 6B), with the significant decrease at 1800 pg/mL (*p* < 0.01). The number and size of lipid droplets in the FGF21 and BML-275 co-treated group exceeded the 900 pg/mL FGF21-treated group visually, but there was little difference between the two in quantitative analysis. For extracellular VLDL (Figure 6C), the results showed that FGF21 significantly promoted the secretion of VLDL from hepatocytes, and the strongest effect was observed with 1800 pg/mL FGF21 compared to no FGF21 treatment (*p* < 0.01). The content of extracellular VLDL was higher in the co-treatment of BML-275 with FGF21 than the 900 pg/mL FGF21 group (*p* < 0.01).

## 4. Discussion

In the periparturient period, dairy cows undergo a state of NEB because of special physiological processes and changes. In such a state, the liver experiences pronounced metabolic stress due to the mobilization of a large amount of NEFAs from the adipose tissue to the blood and liver [23,24]. Under such conditions wherein the oxidation capacity of the liver is exceeded, owing to the presence of excess NEFAs, animals are susceptible to fatty liver disease [3]. The methods for preventing or treating fatty liver disease are as follows: (I) reducing NEFAs in the blood by inhibiting lipolysis in adipose tissue; (II) increasing intrahepatic oxidation of NEFAs; and (III) enhancing the rate of VLDL output from the liver. The disadvantage of the first strategy is that it impedes lactation [25]. Therefore, during the perinatal period, there is a need for appropriate strategies to maintain liver function while ensuring that lactation is unaffected. One possible strategy is to use a treatment that increases liver lipid oxidation and liver lipid transport in order to reduce fat deposition in the liver. As a key mediator of reducing lipid accumulation in bovine liver, FGF21 plays a crucial role in the adaptive regulation of energy metabolism in perinatal cows, which is one candidate for such treatment [26]. Meanwhile, the present study demonstrated that FGF21 induced a reduction in TG levels and increased intracellular VLDL secretion in bovine hepatocytes. FGF21 interacted with a specific receptor complex assembled by the FGFR and β -Klotho proteins, and subsequently activated the AMPK signaling pathway [27,28]. AMPK is activated in order to maintain intracellular energy balance and tissue integrity via the promotion of catabolism and the inhibition of anabolic regulation [12]. LKB1, one of the activators of AMPK, might mediate the activation of AMPK by FGF21. The preferred receptor for FGF21 entry into cells is FGFR1c [27,28]. However, its expression is not high in the liver, so that it may be responsible for the non-linear temporal and dose-dependent effects of FGF21. Caixeta et al. [26] proved that a greater FGF21 content in the blood did not change the mRNA abundance of key genes involved in fatty acid transport, acyl coenzyme A activation, or oxidation. Thus, its physiological role may be overlooked. Fortunately, the present study identified the time-point of peak AMPK activation following FGF21 treatment, and that FGF21 could partially reverse the inhibitory effect of BML-275 on AMPK activity. FGF21 had an effect on the reduction in the intracellular TG and the elevation of extracellular VLDL content for 4 h or 24 h. These results offer the partial possibility that FGF21 improved hepatic lipid metabolism in cows.

The metabolic importance of FGF21 was evident in transgenic mice that overexpressed FGF21 and were deficient in FGF21 [29,30]. Mice overexpressing hepatic FGF21 showed an increased production of liver ketone bodies and an enhanced expression of key genes involved in gluconeogenesis and fatty acid oxidation [9]. In contrast, transgenic FGF21-deficient mice showed reduced rates of gluconeogenesis, β-oxidation, and ketone production during fasting [30]. Thus, the induction of FGF21 is required for the normal activation of liver lipid oxidation, gluconeogenesis, and ketone production in response to nutritional challenges [31]. In rodents, the reduction in liver fat content is mainly caused by increased β -oxidation of fatty acids and reduced de novo synthesis of fatty acids after exogenous FGF21 administration [29]. FGF21 or FGF21 analogues decrease the expression of diet-induced lipogenic genes, including SCD1 FASN and/or SREBF1 [32]. FGF21 significantly increased the expression of CPT1A, PPARD, and PPARGC1A in 3T3-L1 cells [30]. Consistent with mice and humans, the primary tissue for FGF21 synthesis and secretion in dairy cows is the liver [27,29]. During the perinatal period, FGF21 is strongly induced. The up-regulation of gene expression is involved in the growth of fatty acid metabolism, gluconeogenesis, and ketone production in early lactation [31]. Studies have shown that liver and adipose tissue are the main targets of FGF21 from late gestation to early lactation [27]. FGF21 treatment caused a 50% reduction in TG content in the liver [26]. However, super-physiological doses of FGF21 did not affect fatty acid oxidation and synthesis in the liver without altering the mRNA abundance of key genes involved in the transport, acyl coenzyme A activation, or oxidation of fatty acids, because the concentration was 5.8 ng/mL before FGF21 treatment and 179 ng/mL after FGF21 treatment [16,26,27]. These results revealed that FGF21 reduces the translocation of SREBF1 to the nucleus and induces a greater translocation of PPARA to the nucleus. SREBF1 activates gene expressions that control fatty acid and TG synthesis, including ACACA, ACLY, FASN, and SCD1 [33]. PPARA directly regulates the expression of genes involved in fatty acid oxidation in the liver, including ACOX1 and CPT1A [9]. It is important to note that peroxisomes are involved in many lipid metabolic pathways. In ruminants, ~50% of fatty acid oxidation occurs in the mitochondria and the other ~50% occurs in peroxisomes [34]. CPT1 is the key enzyme in this process as it catalyzes the rate-limiting step for the transport of fatty acids into mitochondria and their oxidation [33,35,36]. ACOX1 is the first PPAR target gene to be identified, and it encodes the first enzyme involved in long-chain fatty acid oxidation in peroxisomes [37]. Other studies have shown that SREBF1 overexpression down-regulates the expression and activity of the lipid oxidation enzymes CPT1A and CPT2 in bovine hepatocytes [38]. In the present study, FGF21 affected SREBF1 and PPARA entry into the nucleus through the AMPK signaling pathway. Moreover, it reduced the ratio of ACACA phosphorylation and significantly increased ACLY content, with a significant increase in the mRNA abundance of SCD1 during the co-treatment of FGF21 with the AMPK inhibitor compared to FGF21 alone. The mRNA levels of PPARA, CPT1A, and ACOX1 were increased at that condition. These changes imply that AMPK might play a role in FGF21, affecting the hepatic lipogenesis and lipid oxidation. Although this increased the amount of fatty acid-related proteins synthesized de novo, the inhibition of ACACA activity was more pronounced, i.e., the phosphate ratio was higher than the control group, especially at 900 pg/mL. ACACA activity inhibition reduced malonyl coenzyme A content, removed the inhibition of CPT1A, and increased the mitochondrial fatty acid β-oxidation pathway [35,36]. Therefore, the expression of CPT1A, a key enzyme affecting mitochondrial fatty acid β-oxidation, was also examined in our study in hepatocytes. CPT1A was significantly increased after FGF21 treatment, at least for the mRNA level. Probably, CPT1A protein had a relatively small increase after FGF21 treatment because of the lag in the protein translation process. At the same condition, changes in key proteins of mitochondrial biogenesis showed that SIRT1 and PPARGC1A were increased at least at the transcriptional level after FGF21 treatment, thus increasing the potentiality of mitochondrial biogenesis. In addition to the mitochondrial oxidation key protein, the protein and mRNA levels of ACOX1 were significantly enhanced at low doses. It is possible that fatty acid oxidation in peroxisomes is more susceptible to the effects of FGF21. Therefore, peroxisomal fatty acid oxidation in the liver may need to be further explored in depth during the periparturient period. FGF21 combined with the AMPK inhibitor administration reduced PPARA entry into the nucleus and produced a significant increase in the mRNA levels of PPARA, CPT1A, and ACOX1, compared with FGF21 alone. Therefore, FGF21 could promote fatty acid β-oxidation via AMPK signaling in mitochondria and in peroxisomes.

Fatty acids are the natural ligands of PPARA. L-FABP transports fatty acids to the nucleus and binds to PPAR receptors, activating PPARA, and thus regulating the expression of L-FABP and other PPARA target genes [39,40]. PPARA agonizers enhance the transcription of the L-FABP gene, and PPARA is involved in the up-regulation of fatty acid oxidation via L-FABP. However, excessive fatty acid intake could cause liver fat accumulation. Germline L-FABP deletions reduce the hepatic steatosis caused by high-fat diets, suggesting that L-FABP inhibition may mitigate the effects of hepatic steatosis [41]. Our results also showed a 50% reduction in the protein content of L-FABP after FGF21 treatment, despite an increase in its mRNA abundance. This might be due to post-transcriptional degradation after intracellular lipid oxidation, and there is not much fatty acid to be transported. The role of CD36 in hepatic fatty acid metabolism is not limited to enhancing fatty acid flux and TG accumulation [42]. CD36 deletion aggravates steatosis by impairing the secretion of hepatic TG and ApoB [43]. It has also been found that the FAT/CD36 protein enhances lipid transport into the cell and regulates fatty acid oxidation in a CPT1-independent but AMPK-dependent manner [44]. Our study showed that FGF21 increased the expression of CD36 and the amount of CD36 decreased after inhibiting AMPK activity. When combined with the results of CPT1A, we further confirmed that FGF21 regulated CD36 expression through AMPK signaling pathway and increased the secretion of hepatic TG and ApoB.

TGs in hepatocytes are transported out of the liver through VLDL secretion, reducing TG accumulation in liver. According to clinical and histological studies, three possible mechanisms underlying decreased VLDL secretion in cows with fatty liver disease are as follows: (I) a reduction in VLDL transport from the endoplasmic reticulum to the Golgi apparatus; (II) glycosylation changes in ApoB 100; and (III) a reduction in secretory vesicles [23]. Our study showed that a significant increase in VLDL (approximately 35%) in the supernatant was observed after 900 pg/mL FGF21 treatment for 4 h, indicating that FGF21 stimulated the secretion of VLDL to some extent. ApoB, ApoE, MTTP, and LDLR are the main structural and/or regulatory proteins for the assembly and transport of liver VLDL. Their expression promotes the synthesis and secretion of VLDL from the liver and reduces liver fat content [22,45,46]. ApoE gene deletion results in the blocked synthesis of TG-rich VLDL and fat accumulation in the liver. In contrast, ApoE overexpression increases TG-rich lipoprotein production in the liver [47]. ApoB 100 is usually synthesized and secreted at much higher levels in hepatocytes. Several reports have shown that, in rodents, the secretion of ApoB lipoproteins also increases when free fatty acids and/or TGs are increased in the liver [48,49]. Additionally, the accumulation of ApoB 100 in the endoplasmic reticulum causes ER stress in the liver and disrupts gluconeogenesis, thus promoting the development of insulin resistance in the liver [50]. However, it must be noted that the synthesis of ApoB 100 in cows is lower than that in other animals, and cows thus have a limited ability to secrete VLDL [19]. Therefore, the effect of FGF21 on ApoB 100 in dairy cow hepatocytes might be different from that in rodents, and secretion may be stimulated to a greater extent [51]. Moreover, ApoB binds to TG in order to synthesize lipoproteins, which are transported by MTTP to the lumen side of the endoplasmic reticulum [52,53]. Furthermore, MTTP prevents ApoB degradation and facilitates its assembly into lipoproteins [54]. MTTP-mediated lipid transport inhibitors block ApoB secretion in a concentration-dependent manner [55]. LDLR is not only the endocytosis receptor of VLDL and LDL in the circulatory system, but also promotes the post-translational degradation of ApoB, thereby reducing the secretion of VLDL particles [55,56]. Larsson et al. [57] observed that LDLR inhibited the secretion of ApoB 100 lipoproteins in mice. It was found that the inactivation of LDLR caused an increase in VLDL secretion through an inhibition of ApoB degradation in mice and humans, which resulted in smaller particles being secreted from the liver and decreased TG content. Interestingly, the ability of LDLR to reduce ApoB secretion through reuptake has also been found to be dependent on ApoE expression [56].

In this study, it was found that FGF21 could induce the expression of ApoB 100, MTTP, and ApoE, all of which are assembly components of VLDL. The variations in the levels of these proteins were consistent with the trends observed in VLDL secretion levels (maximum value at physiological dose, likely via AMPK signaling). However, it was shown that the expression of LDLR was higher in FGF21-treated cells than those in the control. Because there was no evidence to suggest that LDLR was a target of SREBF1, we speculated that this difference was because of species- and tissue-level variations [58]. LDLR degradation could have been reduced because FGF21 maintains LDLR stability [59]. However, FGF21 significantly reduced the size and number of lipid droplets in hepatocytes and increased the secretion of VLDL. The point at which AMPK phosphorylation was the highest likely did not correspond with the time-point at which FGF21 exerted the strongest effect on AMPK signaling. Changes in the levels of proteins downstream of AMPK might not be significant, and therefore, no significant differences were observed among the treatment groups. Unfortunately, we did not detect whether there would be an extreme value for the cell uptake of FGF21 to show such results.

**Figure 7:**
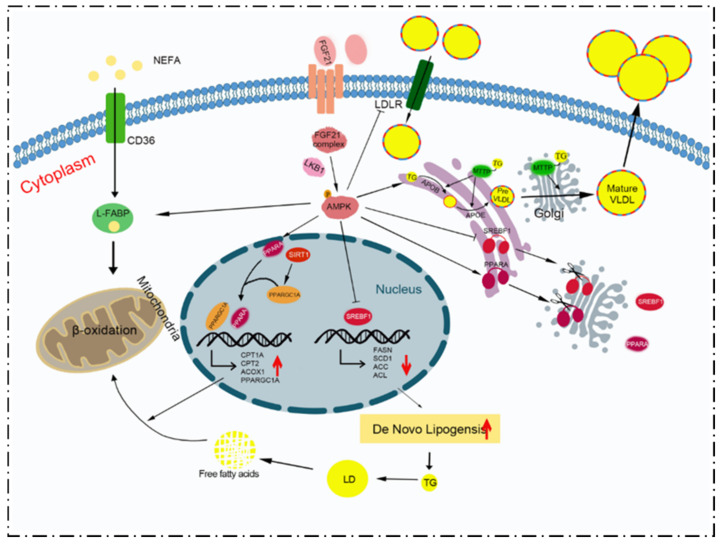
Schematic diagram of the proposed mechanism of FGF21 in regulating lipid metabolism via AMPK signals in bovine hepatocytes. FGF21 mediates AMPK signaling to promote fatty acid oxidation, increases de novo lipogenesis, and promotes TG secretion in the form of VLDL in hepatocytes, ultimately alleviating hepatic lipid accumulation.

Overall, this study has indicated that FGF21 reduces TG synthesis and promotes VLDL secretion in bovine hepatocytes. FGF21 treatment affects two key transcription factors: SREBF1 and PPARA. Under FGF21 treatment, SREBF1 entry into the nucleus, ACLY expression, and ACACA activity are inhibited; however, PPARA is further translocated into the nucleus. The expressions of ACOX1 and CPT1A are promoted. Meanwhile, the present results also suggest that mitochondrial biogenesis might be enhanced after FGF21 treatment due to the increased expression of PPARGC1A. The protein expressions of ApoB 100 and ApoE, which are components of VLDL, are up-regulated by FGF21, as is the expression of CD36 associated with lipid transport. AMPK was found to be partially involved in the process of TG synthesis and metabolism, as well as the assembly and secretion of VLDL (Figure 7). Future research could focus on the role of FGF21 in energy metabolic disorders in perinatal dairy cows, and lay a theoretical basis for exploring new targets and approaches for the prevention and treatment of energy metabolic disorders in dairy cows.

## 5. Conclusions

In summary, this study indicates that FGF21 participates in the adaptive regulation of energy metabolism by reducing TG content and promoting VLDL secretion in bovine hepatocytes. Its mechanism partly involves AMPK signaling, which promotes lipid oxidation and transport in the primary hepatocytes of dairy cows. Additionally, the detailed mechanics need to be explored further in order to provide a strong theoretical support for the prevention and treatment of hepatic lipid metabolism disorders.

## Figures and Tables

**Table 1:** Primer sequences for qRT-PCR.

Gene	Primer Sequences (5′–3′)	GenBank Accession No.
*18S*	For. ACCCATTCGAACGTCTGCCCTATT	NR_036642.1
	Rev. TCCTTGGATGTGGTAGCCGTTTCT	
*SREBF1*	For. CTGACAGCTCCATTGACAAGGC	NM_001113302.1
	Rev. GGCTTCATGTAGGAATACCCTC	
*PPARGC1A*	For. TGCTGCTCTGGTTGGTGAA	NM_177945.3
	Rev. AGGCTCGTTGTTGTACTGATTAG	
*PPARA*	For. ATCAGATGGCTCCGTTATTACAG	NM_001034036.1
	Rev. CAGTATTGGCACTTATTCCGATTC	
*SIRT1*	For. TGTGTCATAGGTTAGGTGGTGAA	XM_024986767.1
	Rev. CTGAAGAATCTGGTGGTGAAGTT	
*ACACA*	For. ATGAAGGCTGTGGTGATGGA	NM_174224.2
	Rev. TGGTGGTCTTGCTGAGTTGA	
*FASN*	For. CAGCGACGTCAGCACACTGGATG	NM_001012669.1
	Rev. GCATGGCATCTCTCAGGACCAC	
*SCD1*	For. CCTGGTGTCCTGTTGTTGTG	NM_173959.4
	Rev. GTGTGGTGGTAGTTGTGGAAG	
*ACLY*	For. GTCAACCTCACTCTGGATGGA	NM_001037457.1
	Rev. TCGTGGTGGAACAGGACATAG	
*CPT1A*	For. GGAATCTGTGAAGCCTCTTATGAA	NM_001304989.2
	Rev. GCCTGGATGTGAGTCGGTAT	
*CPT2*	For. CCTTCCTTCCTGTCTTGGTATG	NM_001045889.2
	Rev. TTCAGAGGCACTCACAATGTTC	
*ACOX1*	For. TAAGCCTTTGCCAGGTATT	NC_015500.1
	Rev. ATGGTCCCGTAGGTCAG	
*CD36*	For. CTCATTGCTGGTGCTGTCATT	NM_001278621.1
	Rev. CCTTGGCTAGATAACGAACTCTG	
*LDLR*	For. AATGCGAGTGTGAAGAGG	XM_010806777.3
	Rev. GGTGTCGTAGGAGGAGAA	
*L-FABP*	For. GGAGGAGTGTGAGATGGAGTT	NM_175817.3
	Rev. CCTTCGTCATGGTACTGGTAAC	
*ApoB 100*	For. GAACAGAATGAGCAAGTGAAGAAC	XM_024999521.1
	Rev. AGGTCAAGTGATGGCAGAGAA	
*ApoE*	For. GCCGCTTCTGGGATTACCT	NM_173991.2
	Rev. GGTTCCGCAAGTCCTCCAT	
*MTTP*	For. GAACAGGATATACCACCAGAATCG	NM_001101834.1
	Rev. CTTCAGAACTTGACGGACCATT	

## Data Availability

The raw data supporting the conclusions of this article will be made available by the authors, without undue reservation.
